# Cold-Induced Reversible Cerebral Vasoconstriction Syndrome Presenting With Intraventricular Hemorrhage in a Normotensive Patient

**DOI:** 10.7759/cureus.87238

**Published:** 2025-07-03

**Authors:** Layla M Babiker, Ahmed I Idriss, Yousif Yousif

**Affiliations:** 1 Internal Medicine, Sudan International University, Khartoum, SDN; 2 Internal Medicine, University of Khartoum, Faculty of Medicine, Khartoum, SDN; 3 Neurology, Jazan University, Jazan, SAU

**Keywords:** cerebral vasospasm, cold-induced rcvs, intraventricular hemorrhage, normotensive intracerebral hemorrhage, reversible cerebral vasoconstriction syndrome (rcvs), thunderclap headache

## Abstract

Reversible cerebral vasoconstriction syndrome (RCVS) is a transient neurovascular disorder characterized by sudden, severe headaches and reversible multifocal cerebral artery narrowing. While it is often associated with vasoactive substances, emotional stress, or postpartum status, environmental triggers such as cold exposure are rarely reported. We describe the case of a 61-year-old normotensive female who developed a thunderclap headache and vomiting shortly after brief exposure to cold weather. Initial evaluation revealed hypothermia and metabolic acidosis; she was discharged without neuroimaging. Three days later, brain imaging revealed a subacute left caudate intracerebral hemorrhage with intraventricular extension. Her condition deteriorated following an acute hypertensive episode attributed to anxiety. CT angiography and digital subtraction angiography demonstrated multifocal cerebral vasospasm without aneurysm or arteriovenous malformation. A working diagnosis of RCVS was made, and the patient improved with calcium channel blocker therapy (nimodipine) and supportive care. This case emphasizes the importance of recognizing RCVS in hemorrhagic presentations, even in normotensive patients, and suggests a potential role of cold-induced autonomic dysregulation in its pathogenesis.

## Introduction

Reversible cerebral vasoconstriction syndrome (RCVS) is an underrecognized yet increasingly reported neurovascular condition characterized by sudden-onset severe headaches - often described as thunderclap in nature - and reversible segmental narrowing of the cerebral arteries. It is typically self-limiting but can lead to significant complications, including ischemic stroke, subarachnoid hemorrhage, and, less commonly, deep intracerebral hemorrhage [1,2].

The pathophysiology of RCVS is believed to involve transient dysregulation of cerebral vascular tone, driven by sympathetic overactivity or endothelial dysfunction [3]. Known triggers include physical exertion, emotional stress, exposure to vasoactive substances (such as selective serotonin reuptake inhibitors or sympathomimetics), and postpartum states. However, environmental factors such as cold exposure are rarely reported as precipitants in the literature, making such cases valuable for expanding clinical awareness [2,4].

Diagnosing RCVS can be challenging due to its clinical overlap with other serious conditions such as primary angiitis of the central nervous system (PACNS), aneurysmal subarachnoid hemorrhage, and hypertensive encephalopathy. While imaging findings such as multifocal segmental vasoconstriction on CT or MR angiography are helpful, the diagnosis often requires a combination of clinical history, exclusion of structural lesions, and radiographic reversibility of vasospasm [5,6].

This case report presents a previously normotensive 61-year-old woman who developed a caudate nucleus intracerebral hemorrhage with intraventricular extension shortly after a brief episode of cold exposure. The clinical course and imaging findings were most consistent with RCVS, supported by the presence of multifocal vasospasm, absence of vascular malformation, and favorable response to calcium channel blockade. This case is notable for its rare environmental trigger, the presence of deep hemorrhage in the absence of chronic hypertension, and the potential contribution of emotional stress and selective serotonin reuptake inhibitor (SSRI) use. It also underscores the importance of maintaining a high index of suspicion for RCVS in atypical hemorrhagic presentations.

## Case presentation

A 61-year-old female with a background of prediabetes (on metformin), sertraline use for menopausal symptoms, and intermittent corticosteroid use for food-allergy-related headaches presented with a sudden, severe headache - described as the worst of her life - after a brief 5-7-minute walk outdoors in cold weather. The headache was associated with five episodes of vomiting. She had no history of hypertension, smoking, alcohol use, or anticoagulant/antiplatelet medications.

She initially presented to a local emergency department, where she was diagnosed with hypothermia (temperature unrecordable), metabolic acidosis (pH 7.30, lactate 5.8 mmol/L, anion gap 18), and was treated with IV fluids and warming blankets. No neuroimaging was performed, and she was discharged home. This represents a missed opportunity, as early neuroimaging could have identified the hemorrhage and expedited intervention.

Three days later, a non-contrast CT brain performed at a private clinic revealed a small (3-7 mm) left caudate intracerebral hemorrhage (ICH) with intraventricular extension, estimated to be subacute. Although clinically stable, an MRI of the brain was planned for further evaluation. While awaiting imaging results, she experienced acute anxiety, followed by rapid clinical deterioration marked by severe headache, vomiting, and confusion. Her blood pressure was elevated at 200/127 mmHg, for which she received IV labetalol. This abrupt rise in blood pressure may reflect sympathetic overdrive and cerebral autoregulatory dysfunction, consistent with RCVS pathophysiology.

Upon transfer to a tertiary neurosurgical center, repeat CT imaging demonstrated interval reduction in hemorrhage density within the left caudate and internal capsule, with a persistent large-volume intraventricular hemorrhage and stable mild hydrocephalus. No new parenchymal, extra-axial, or osseous abnormalities were noted. The patient was post-digital subtraction angiography (DSA) at the time of this scan.

**Figure 1:**
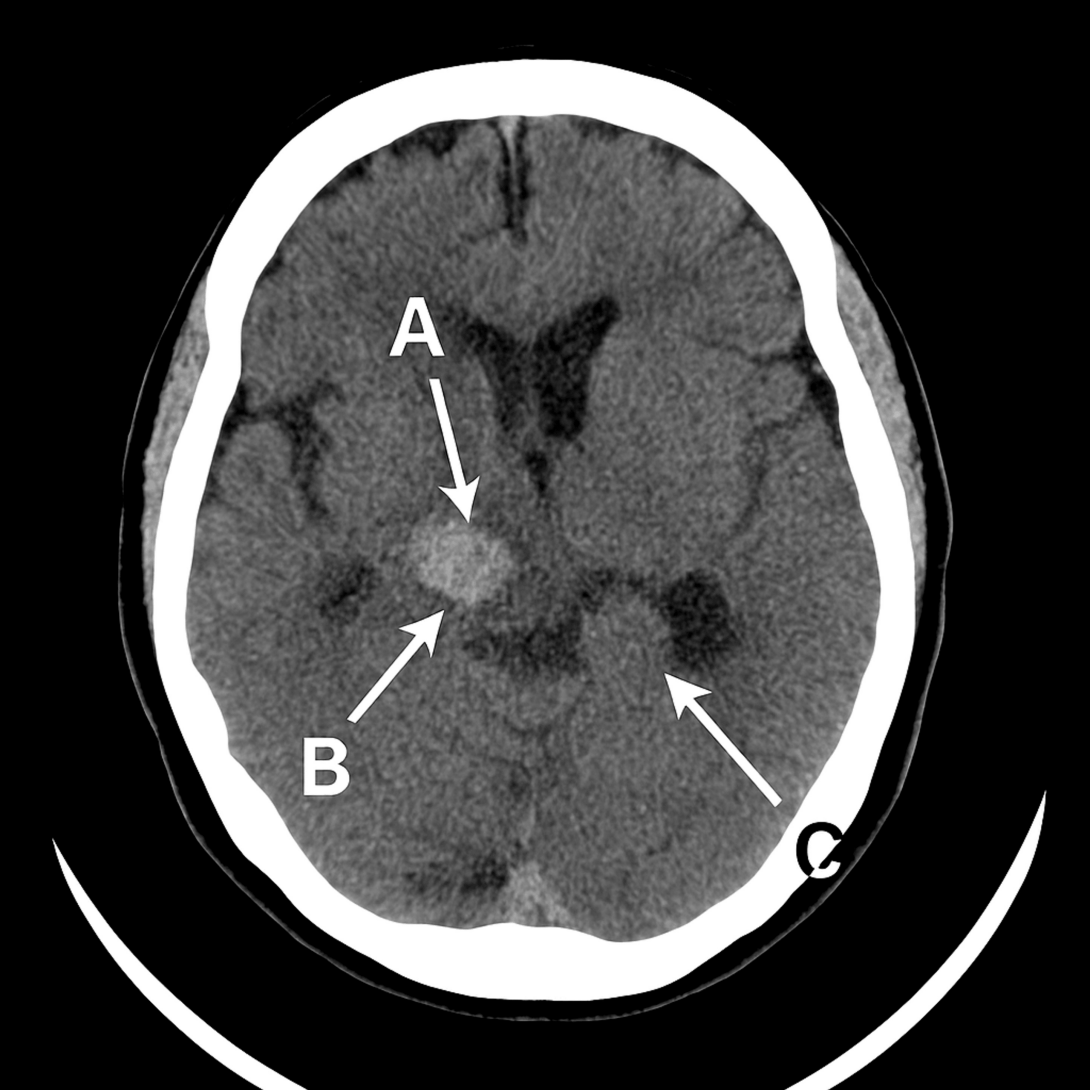
Axial non-contrast CT image of the brain demonstrating a stable acute hematoma (arrow A) in the left caudate nucleus, with intraventricular extension (arrow B). There is also mild prominence of the temporal horns (arrow C), consistent with communicating hydrocephalus. No evidence of infarction, trans-ependymal edema, or further hemorrhage is noted. A mild local mass effect is present.

A subsequent non-contrast CT stealth scan was performed for surgical planning, given the worsening neurological status (Glasgow Coma Scale (GCS) drop from 15 to 10). Imaging showed a stable acute hematoma with persistent intraventricular extension, slight prominence of the temporal horns, and mild communicating hydrocephalus, with no signs of transependymal edema, infarction, or new hemorrhage (Figure 1).

**Figure 2:**
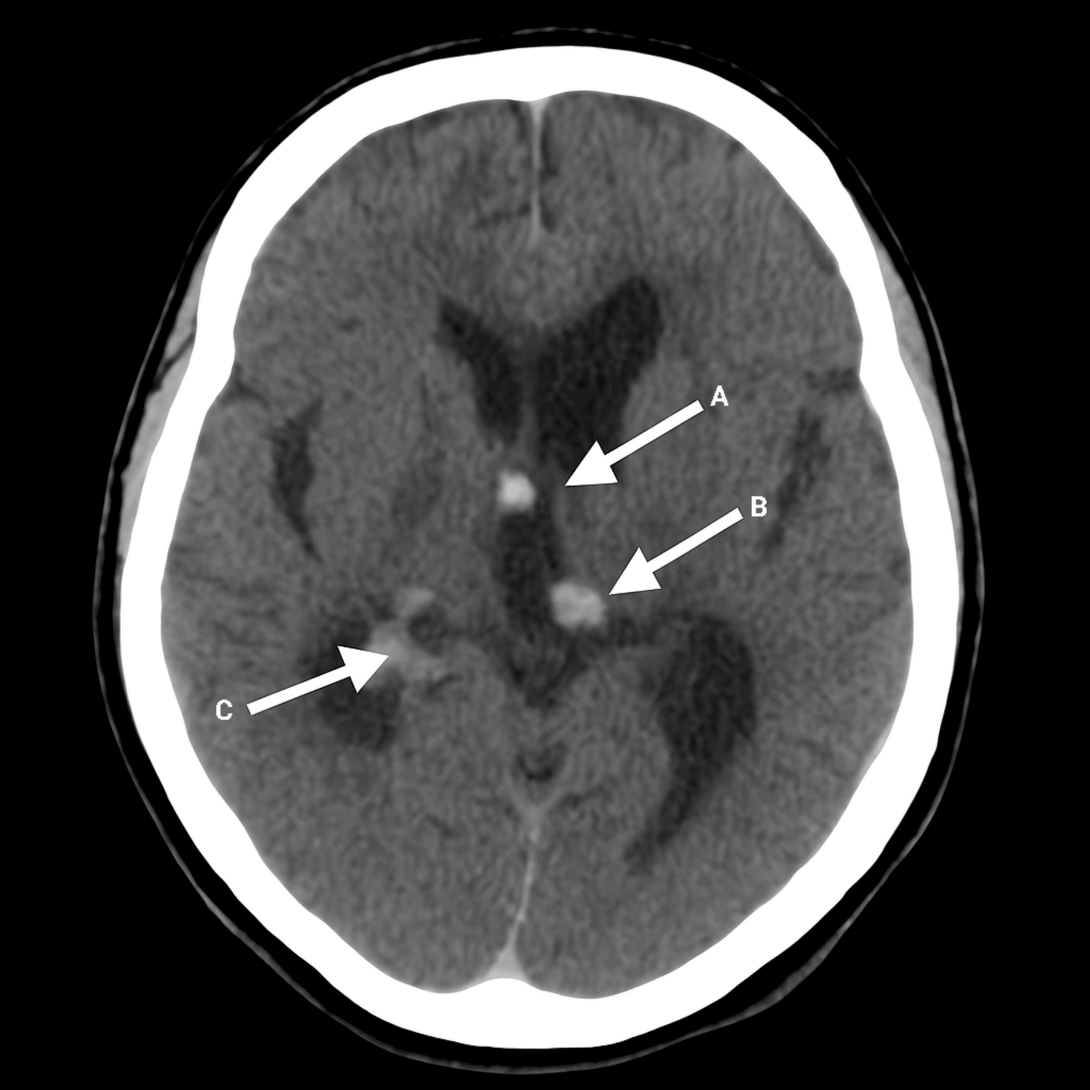
Axial Non-contrast Stealth CT Brain – Stereotactic Planning Prior to CSF Diversion. This axial CT image demonstrates a large acute intracranial hematoma centered in the left caudate nucleus (Arrow A), with intraventricular extension visible in the lateral and third ventricles (Arrow B). There is associated mild surrounding edema. Probable mild communicating hydrocephalus is suggested by prominence of the temporal horns (Arrow C). No evidence of transependymal edema or vascular malformation is noted. Moderate arterial narrowing was later confirmed on angiography, consistent with suspected vasospasm.

CT angiography demonstrated moderate vasospasm in the right middle cerebral artery (MCA) M2 segment and bilateral posterior cerebral arteries (PCAs), without evidence of aneurysm or arteriovenous malformation. These findings, particularly in the absence of aneurysm or arteriovenous malformation (AVM), are strongly suggestive of RCVS. DSA later confirmed multifocal vasospasm in the left MCA M1-M3 segments, with no evidence of AVM, fistula, or aneurysm (Figure 2).

Neurology and stroke services deemed the most likely diagnosis to be RCVS, though workup for PACNS was initiated for exclusion. Lumbar puncture was planned to assist in differentiation. Blood work, including autoimmune and vasculitis panels, was sent.

She was started on oral nimodipine and oxycodone for severe headache, with notable improvement in pain control. Over the following days, her clinical status improved significantly. She became fully oriented (GCS 15), hemodynamically stable (BP ~120/75 mmHg on amlodipine), and reported resolution of headaches and restoration of appetite. She was able to record a video message expressing gratitude to her family - reflecting meaningful functional recovery.

Follow-up imaging showed no new hemorrhage or hydrocephalus. No echocardiogram or fundoscopy was performed. The lack of these evaluations may have missed other potential causes of intracranial hemorrhage or systemic contributors. A 24-hour ambulatory BP monitor was arranged to assess for occult hypertension, which returned normal values.

## Discussion

RCVS is a clinical and radiographic syndrome characterized by recurrent thunderclap headaches, reversible segmental vasoconstriction of cerebral arteries, and, in some cases, associated hemorrhages or infarctions [1]. While classically associated with postpartum states, vasoactive substances, and emotional or physical stressors, RCVS triggered by cold exposure is rare and not well-documented in the literature [2].

This case presents a likely instance of RCVS precipitated by hypothermia, compounded by stress-induced hypertensive episodes in a previously normotensive patient. The initial presentation - sudden severe headache and vomiting after cold exposure - raises concern for a vascular insult such as RCVS, subarachnoid hemorrhage, or intracerebral hemorrhage [3].

Subsequent CT revealed a left caudate hemorrhage with intraventricular extension. Caudate location is common in hypertensive ICH, yet this patient had no history of hypertension. Instead, transient hypertensive spikes - likely stress-related - were documented during deterioration, particularly around MRI anxiety. Such spikes may reflect sympathetic overactivation or cerebral autoregulatory dysfunction, mechanisms implicated in RCVS pathophysiology [4].

CT angiography and DSA confirmed segmental vasospasm in multiple territories, including the MCA and PCAs. These findings, combined with gradual symptom resolution on nimodipine, strongly support RCVS [5]. The absence of aneurysm, AVM, or fistula further supports this diagnosis.

The use of sertraline, an SSRI, adds a potential precipitating factor, as SSRIs have been associated with RCVS in previous reports [6]. Intermittent corticosteroid use may complicate interpretation; while steroids are used in PACNS, they are not recommended in RCVS and may worsen vasoconstriction [7].

The key differential diagnosis is primary angiitis of the CNS (PACNS). Unlike RCVS, PACNS tends to present with insidious onset, non-reversible vasculopathy, and a greater likelihood of infarcts than hemorrhage [8]. In this case, the acute onset, reversible angiographic findings, and clinical improvement favor RCVS.

RCVS-related deep hemorrhage is uncommon, and its occurrence in a normotensive patient following cold exposure makes this case unique. Notably, Kanani et al. reported autopsy evidence of sudden and fatal nontraumatic brain hemorrhages in individuals without prior hypertension or known medical history, further highlighting the need for vigilance in such atypical presentations [9]. The lack of fundoscopy, echocardiography, or CSF analysis at initial deterioration reflects real-world diagnostic limitations but does not detract from the clinical impression supported by imaging and course.

## Conclusions

This case highlights an unusual presentation of RCVS triggered by environmental cold exposure, accompanied by acute intracerebral hemorrhage in a normotensive patient. It underscores the importance of maintaining clinical suspicion for RCVS even in hemorrhagic cases and in patients without traditional risk factors such as chronic hypertension. Additionally, the case illustrates how stress-induced hypertensive episodes and serotonergic medication may contribute to vascular dysregulation. Prompt recognition, supportive care, and the use of calcium channel blockers such as nimodipine were associated with favorable clinical recovery. Clinicians should consider RCVS in the differential diagnosis of thunderclap headache with hemorrhagic findings, especially when initial imaging reveals vasospasm and structural vascular lesions are excluded.
